# A Possible Treatment for Persistent Cough Status Post-pulmonary Carcinoid Tumor Resection

**DOI:** 10.7759/cureus.25499

**Published:** 2022-05-30

**Authors:** Gedaliah May, Micah M May

**Affiliations:** 1 Cardiothoracic Surgery, Touro College of Osteopathic Medicine, New York, USA; 2 Internal Medicine, Micah M May MD, Lakewood, USA

**Keywords:** chronic cough, pulmonary resection, acupuncture, persistent cough, post-surgical cough

## Abstract

Cough is a common symptom of many underlying pathologies; sometimes, the etiology is well understood and, therefore, treatment can be applied accordingly. However, when the underlying etiology of the cough is not well understood, a nonconventional approach can sometimes be promising. In this article, a cough of unknown etiology resistant to conventional treatments seems to be suppressed with the use of acupuncture techniques.

## Introduction

The ability to cough plays an essential role in keeping our lungs clean. Coughing allows the body to expel foreign material from our lungs as a protective factor. The cough reflex is complex, involving afferent signals from the vagus nerve and its branches and efferent signals through the phrenic nerve, vagus nerve and its branches, and more. These afferent neurons synapse in the brainstem and relay information between the cerebral cortex and other brain centers, which finally terminate as an efferent signal that produces a cough [1]. Typically, when receptors located in the respiratory tract are stimulated by mechanical or chemical irritants, a cough is produced [1]. Sometimes the cough reflex can be produced with stimulation elsewhere along the cough reflex neural pathway (e.g., Arnold's reflex) [2].

In some pathological situations, the cough reflex can be stimulated as a protective factor; however, sometimes, a pathological condition can elicit a cough gratuitously. A carcinoid tumor is a rare neuroendocrine tumor most commonly found in the gastrointestinal tract or in the lungs [3]. Surgical resection is sometimes preferred when a carcinoid tumor is found in the lung [4]. A common symptom of an underlying carcinoid tumor is a cough. Furthermore, when surgical intervention is indicated for a carcinoid tumor, a post-surgical cough is a common finding as well [1].

In the case of a pulmonary carcinoid tumor, the occurrence of cough is multifactorial owing to either tumor tissue compression, inflammation, or airway blockage leading to cough receptor stimulation [5]. Additionally, carcinoid tumors can secrete hormones, most notably, serotonin causing bronchoconstriction, which may present as wheezing or cough [6]. Here, we will discuss an interesting case where a persistent chronic cough with an unknown etiology resistant to prescription and over-the-counter medications was successfully suppressed with the use of acupuncture.

## Case presentation

**Figure 1:**
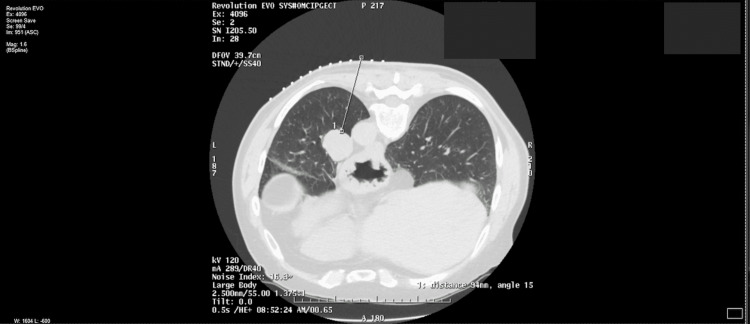
Computed tomography scan showing pulmonary carcinoid tumor prior to surgical resection

A 77-year-old Caucasian male presented for a follow-up visit three weeks after undergoing a left lower lung lobectomy and lymph node resection for a biopsy-proven carcinoid tumor (Figure 1). The patient was doing well postoperatively, except for post-surgical cough. The patient had a history of chronic cough preoperatively along with a chronic complaint of rhinorrhea and occasional sneezing that prompted further diagnostic testing and eventual diagnosis. However, the patient describes this current cough to seem a bit different than his pre-surgical cough, stating that it is worse when he talks and lies down.

Ten days after lobectomy, the chest radiograph was clear and unequivocal. The patient denied fevers, chills, night sweats, sputum production, sore throat, chest pain, hemoptysis, and dyspnea. On physical exam, the patient's vitals were within normal limits with an oxygen saturation of 97% on room air. The patient's lungs were clear on auscultation with no abnormal breath sounds.

The patient tried using over-the-counter dextromethorphan cough medication without success. A trial of montelukast sodium 10 mg tablet once a day at bedtime was started, which mildly reduced the amount of coughing as reported by the patient. However, a few weeks later, the coughing was still excessive, and benzonatate as 100 mg capsule for every eight hours was added. The addition of benzonatate did not seem to reduce the coughing to a tolerable amount. The patient estimated that he coughed at least 50 times per hour. It was worse when he spoke, making it difficult to socialize or talk on the phone. His coughing was worse when lying in a supine position causing him great nocturnal distress. Overall, this cough contributed to a reduced quality of life for this patient.

After some internet research on the treatment of postoperative cough done by the patient’s wife, who was a registered nurse, acupuncture seemed to be a good idea to investigate further. The patient contacted a registered acupuncturist in Florida who requested the patient to fill out a medical questionnaire and submit a photograph of the patient's tongue before scheduling an appointment for acupuncture therapy. After review, the licensed acupuncturists called the patient to discuss the cough, overall medical condition and history, her analysis of the patient's tongue, and how acupuncture can help suppress the cough. Initially, the acupuncturist recommended a total of 10 weekly treatment appointments which the patient began. However, the patient stopped after four sessions because he no longer suffered from severe coughing. Two weeks after his last acupuncture treatment, he stopped coughing excessively. The patient claimed a 90% reduction in coughing.

## Discussion

Cough is a common symptom among many patients who have undergone lung surgery, and management can be challenging. The etiology of a post-surgical cough is ill-defined. Although there have been a few proposed mechanisms, no consensus has been reached, hence the need for further investigation [1]. In their systematic review [1], Li et al. described in depth many of the currently proposed etiologies of a post-surgical cough. In this study, we have highlighted the ideas that pertain to our case.

Trauma, inflammation, and scar formation in lung tissue and nerves in the vicinity secondary to the surgical procedures are thought to play a role in irritating excessive cough. Additionally, foreign bodies such as sutures may play a similar role as well [1]. Cough receptors are mainly located in the larynx, trachea, carina, and large bronchi. Therefore, lymph node resection especially mediastinal lymph nodes which are in close proximity to these receptors are thought to be another cause of post-surgical cough [1].

The anatomical and physiological balances can be disturbed in lung resection procedures as in our case of left lower lung lobectomy. The removal of lung tissue results in hemithoracic volume loss leading to a decreased intrathoracic pressure. The body will compensate initially, but later on, this can lead to an increased respiratory effort, airway sensitivity, and persistent cough [1].

Currently, there are many antitussives or cough remedies. None of these pharmacological agents were successful in our case, hence the trial of acupuncture sessions. The patient reported a 90% decrease in coughing just after two weeks of acupuncture therapy. Although a relationship might exist between acupuncture and reduced cough severity, there is not enough evidence from this case alone to suggest that acupuncture was the sole reason for the reduction of this patient's cough. Therefore, clinical judgment should be used to guide management. This case report is intended only for educational purposes.

A brief synopsis of acupuncture is discussed in this report [7]. Acupuncture was one of the oldest medical procedures in the world dating back to approximately 2000 years, which was originated in China. Over the years, it has diversified to encompass many styles, techniques, and specialties. The most commonly practiced techniques involved mechanical or electrical manipulation of fine needles inserted into precisely defined points on the skin. These points are located along 14 main channels that connect the body and correlate with specific organs not necessarily related anatomically. A typical session involves the insertion of five to 20 needles left in place for a few minutes, while the patient relaxes and then removed. Treatments can last between 15 and 60 minutes and occur one or two times a week. A survey done in 2012 estimated that 3.8 million US adults had used acupuncture within the last year [2]. Common conditions treated with acupuncture were back, neck, and joint pain along with headaches.

There are some proposed mechanisms of action as to how acupuncture works. However, the data is either inconsistent or inadequate to arrive at a conclusion. Here are some hypotheses: Needle stimulation causes the release of endorphins leading to the analgesic effects seen with acupuncture treatment [2]. Another theory is based on MRI studies that showed an association between acupuncture and blood oxygenation level-dependent signals in the central nervous system. These changes were seen as a delayed and long-sustained increase and decrease of blood oxygenation levels in the somatosensory region and in areas related to pain perception [2]. There is a third theory that acupuncture needles inserted in areas of loose connective tissue cause the collagen to twist. This mechanical force is hypothesized to contribute to the analgesic effects by influencing local purinergic and inflammatory signaling pathways [2].

## Conclusions

A common sequela of postoperative lung surgery is cough. The etiology of this cough is not well understood, and there are many proposed theories. Management can be challenging especially when the cough seems to be resistant to conventional therapies. This case illustrated the usefulness of acupuncture for post-lung surgery cough. Our patient presented with a persistent severe cough post left lower lung lobectomy for carcinoid tumor. Following the failure of response to the usual antitussives, our patient underwent acupuncture therapy. He reported a 90% decrease in his coughing just after four acupuncture sessions. This improved his quality of life, and he was able to return to his normal daily living. 

Although our case report might reflect a probable link between acupuncture and cough improvement, larger studies are needed before any association can be established. A comparative study using an antitussive versus acupuncture or an add-on to antitussives for resistant cough would be helpful. However, there is insufficient evidence from this case alone to suggest that acupuncture was the sole reason for the reduction of this patient's cough. Therefore, clinical judgment should be used to guide management. This case report is intended only for educational purposes.
